# Volatile fatty acids recovery from thermophilic acidogenic fermentation using hydrophobic deep eutectic solvents

**DOI:** 10.1186/s13036-025-00544-6

**Published:** 2025-07-31

**Authors:** Can Liu, Xueyao Zhang, Qi Qiao, Zhiwu Wang, Qing Shao, Jian Shi

**Affiliations:** 1 Department of Biosystems and Agricultural Engineering, University of Kentucky, Lexington, KY 40546 USA; 2 Department of Biological Systems Engineering, Virginia Polytechnic Institute and State University, Blacksburg, VA 24061 USA; 3 Department of Chemical and Materials Engineering, University of Kentucky, Lexington, KY 40506 USA

**Keywords:** Volatile fatty acid, Arrested methanogenesis, Membrane extraction, Deep eutectic solvent, Molecular simulation

## Abstract

**Background:**

Volatile fatty acids (VFA) derived from acidogenic fermentation can be recovered as precursors for synthesizing value-added chemicals to replace those from fossil fuels. However, separating VFAs from the fermentation broth with complex constituents and a high-water content is an energy-intensive process.

**Results:**

This study developed an innovative membrane extraction technology, utilizing hydrophobic deep eutectic solvents (HDESs) as the acceptor phase along with an omniphobic membrane contactor for efficient extraction of anhydrous VFAs. All tested HDESs, three terpene-based type V HDESs and two tetraalkylammonium halide-based type III HDESs, were found to effectively extract VFAs at pH 3, with extraction recovery percentages (ERPs) up to 80% and 92% for 4 C- and 5 C- VFAs, respectively. However, the ERP of type V HDESs decreased significantly when the aqueous phase was adjusted to pH 6. Molecular simulations suggest that the VFA-HDES interactions vary with VFA dissociation, where the ion-dipole interactions between VFA conjugate bases and hydrogen bond donors at near-neutral pH conditions may destabilize the type V HDES structure and lead to reduced extraction efficiency. The temperature increases from 25 °C to 55 °C did not significantly impact VFA distribution, but a higher temperature could enhance cross-membrane mass transfer.

**Conclusions:**

This study demonstrated a novel continuous VFA extraction technology based on HDESs and elucidates the impact of temperature, pH, impurities in real fermentate and the applicability of an integrated membrane system through combined experimental and computational approaches.

**Supplementary Information:**

The online version contains supplementary material available at 10.1186/s13036-025-00544-6.

## Introduction

Acidogenic fermentation (AF) is an effective approach for stabilizing organic wastes while generating value-added products, particularly volatile fatty acids (VFA) [1]. As the initial phase of the well-established anaerobic digestion (AD) process, AF can be enhanced by increasing the process temperature (thermophilic AD) or by increasing the organic loading rate. These modifications often lead to a lower pH, which arrests the subsequent methanogenesis step and allows the process to conclude at the acidogenesis stage [2, 3]. However, the accumulation of VFAs during AF can inhibit further fermentation through product inhibition, ultimately limiting VFA yields [4]. To address this issue and establish an effective, continuous, high-yield VFA production system, it is essential to implement in-situ separation and recovery of VFAs from the fermentation broth.

VFAs recovery remains a major technical challenge, despite the investigation of several methods — including gas stripping, adsorption, solvent extraction, electrodialysis, and various membrane-based separation — to separate VFAs from the complicated constituents of AD systems [5]. For example, in solvent extraction, the commonly used extractants for VFAs are typically dissolved in some toxic diluents such as alcohols (C5 ~ C12) and ketones (C5 ~ C8), which could lead to the failure of AF process even if a trace amount was transferred to the aqueous phase (digestate) [6, 7]. Conventional membrane-based separation methods, on the other hand, typically rely on external energy input to create a pressure or temperature differential between the donor and acceptor phases, so as to supplement the limited driving force from the solute concentration gradient and facilitate the transfer of VFAs across the membrane [8, 9]. To address this limitation, a promising strategy is to combine liquid–liquid extraction with membrane-based separation techniques. In this system, the affinity of VFAs for the extractant enhances the driving force for their transfer, while the membrane serves to prevent the diffusion of extractants into the aqueous phase and to reject pollutants present in the fermentate [10, 11]. For instance, Aydin et al. developed a trioctylamine (TOA)–filled PTFE membrane contactor for continuously separation of VFA from AD reactor [12]. In such a system, TOA, acting as a Lewis base, showed high effectiveness extracting carboxylic acids [13].

Conventional carboxylic acid extractants, such as alkylamines and phosphine oxides, are typically used with active diluents to enhance the properties of the acceptor phase [14]. However, these diluents can suppress interactions between the extractant and VFAs, and may also introduce environmental and economic drawbacks [15]. Furthermore, conventional extractants are generally effective only for extracting undissociated VFA molecules, and their performance declines under basic conditions [15, 16]. To address these limitations, recent studies have explored the use of hydrophobic ionic liquids (ILs) and type III hydrophobic deep eutectic solvents (HDESs) based on quaternary phosphonium or ammonium salts. These solvents can recover VFAs through ion exchange and hydrogen bonding between their anions and both dissociated and undissociated carboxylic acids [17–19]. Notably, they can function across a range of pH conditions due to their dual affinity for both forms of the acid. For example, the hydrophobic IL [P_666,14_][Phos] has been shown to efficiently extract VFAs from artificial fermented wastewater at pH 5.0 [18].

Building on IL frameworks, Type III HDESs retain quaternary cationic centers but replace IL anions with hydrogen-bond donors, resulting in reversible hydrogen-bond networks that improve biodegradability, lower toxicity, and simplify preparation while maintaining high extraction efficiency [15, 17]. In addition, type V HDESs, which are composed of economical and environmentally friendly natural terpene ingredients, are believed to possess comparable efficiency in extracting undissociated VFAs through intermolecular interactions [20, 21]. This property makes them suitable for use in combination with membrane techniques, which generally allow only vaporized VFA molecules to permeate. However, the effects of pH and temperature on the extraction process of type III and type V HDESs remain unclear, due to a limited understanding of their extraction mechanisms.

In this study, we investigated the extractability of five HDESs for extracting VFAs from a synthetic aqueous solution under various temperature and pH conditions. Based on their desirable stability, hydrophobicity and extraction efficiency, we selected two tetraalkylammonium halide-based type III HDESs — TBAC/DA (1:2) [22] and TOAB/DA (1:2) [17] — as well as three terpene-based type V HDESs — Men/Thy (1:1) [23], Men/DA (2:1) [24], Thy/DMP (1:2) [25]. The numbers indicate molar ratios and Men, Thy, DA, DMP, TBAC and TOAB denote menthol, thymol, decanoic acid, 2,6-dimethoxyphenol, tetrabutylammonium chloride and tetraoctylammonium bromide, respectively. Molecular simulations for representative type III and V HDES systems were also conducted to investigate how temperature and pH affect their extraction performance. To determine the feasibility of these HDESs in dealing with real fermentate through the above-proposed direct or membrane-assisted extraction, effluent from a laboratory thermophilic AD system was applied in (1) a direct extraction test with a type III HDES and (2) a membrane-assisted extraction experiment using a membrane contractor with a type V HDES. This study demonstrates a novel continuous VFA extraction technology enabled by HDESs and elucidates the impact of temperature, pH, and impurities on the integrated membrane system using a combined experimental and computations approach.

## Methods

### Materials

Acetic acid (> 99.7%) and valeric acid (99%) were supplied by BeanTown Chemical. Butyric acid (≥ 99%), propionic acid (≥ 99.5%), isobutyric acid (99%), Men (≥ 99%), Thy (≥ 99%), DA (≥ 98%), DMP (≥ 98%), TBAC (≥ 97%) and TOAB (≥ 98%) were purchased from Sigma-Aldrich. Ammonia solution (30%) and hydrochloric acid (10% v/v) were obtained from VWR Chemicals. All chemicals were used as received without further purification. Deionized water was used in all experiments. The effluent from a thermophilic acidogenic fermentation of brewer’s spent grains at 55 °C [26] was used to represent real fermentate. Prior to extraction tests, the effluent was filtered through 0.45 μm nylon syringe filters (VWR International, catalog no. 76479-032).

### HDES preparation and VFA solution preparation

**Fig. 1:**
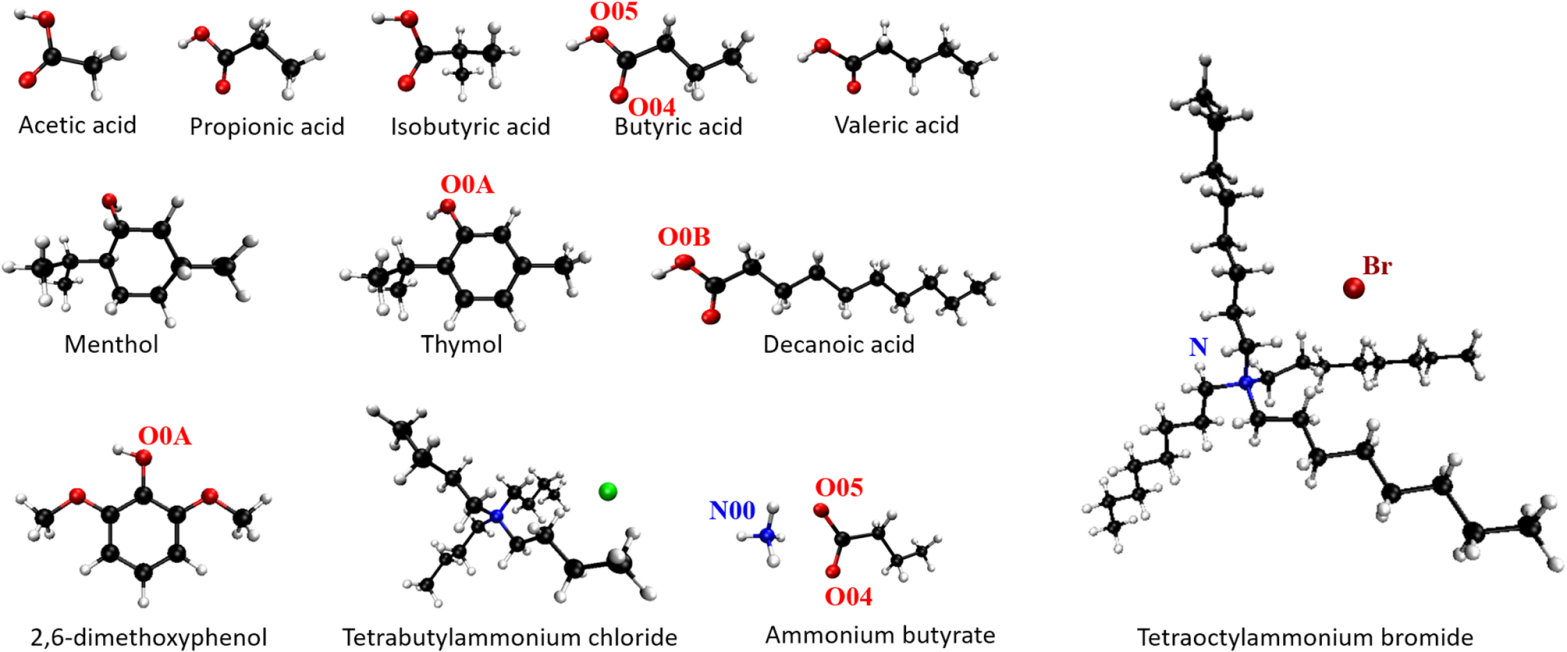
Molecular structures of the tested VFAs and HDES-forming components, shown in the CPK model (C: black, O: red, H: white, N: blue, Cl: green, Br: dark red). Atoms used for radial distribution function analysis in MD simulations are labeled

The molecular structures of the VFAs of interest and HDES-forming components are shown in Fig. 1. The characteristics of the aqueous phases in extraction experiments are shown in Table 1 with each condition abbreviated based on its composition (*S*,* A*,* F* series) followed by its pH condition (*-a* or *-n*). A synthetic mixture (*S* series) with a fixed concentration of 10 mM for various VFAs was utilized to investigate the effect of pH and temperature on the extraction performance in general. Ammonia solution (10%) or hydrochloric acid (1 N) was used to adjust the pH of the aqueous phase to pH 3 for acidic (*-a*) or pH 6 for neutral (*-n*) conditions, respectively. The filtered effluent from a real fermentate (*F* series) as described above was utilized as the VFA-rich aqueous phase. The VFAs composition in the effluent was monitored for the first 4 days of the fermentation process. Based on this data, an artificial fermentate (*A* series) was prepared by setting the concentration of each VFA species to its highest level achieved throughout the 4-day fermentation process. The *A* series contained only the five VFAs and ammonia, with no additional components, ensuring a controlled comparison. This artificial fermentate was used as a substitute for real fermentate in some preliminary experiments and served as a control to evaluate the effect of impurities present in the real effluent.

**Table 1:** Aqueous solution preparation for direct or membrane extraction experiments

Conditions ^a^	pH	Adjusted with	VFA concentrations (mM)				
			Acetic acid	Butyric acid	Propionic acid	Isobutyric acid	Valeric acid
*S-a*	3	N/A	10	10	10	10	10
*S-n*	6	NH_4_OH (10%)	10	10	10	10	10
*A-n*	6	NH_4_OH (10%)	255	120	41	4	3
*F-n*	6	N/A	132 ~ 255 ^b^	104 ~ 120 ^b^	38 ~ 41 ^b^	0.8 ~ 4 ^b^	1 ~ 3 ^b^
*F-a*	3	HCl					

^a^ S, A, F, -a, and -n stands for Synthetic solution, Artificial fermentate, Filtered fermentate, acidic pH and neutral pH, respectively

^b^ In F series conditions, a real effluent from the thermophilic acidogenic fermentation system was used, and the VFAs concentrations vary with fermentation conditions

Five different HDESs were tested as extraction solvents: Men/Thy (1:1), Men/DA (2:1), Thy/DMP (1:2), TBAC/DA (1:2) and TOAB/DA (1:2), with compositions and abbreviations as defined in the Introduction section. Each DES was prepared by mixing the two components in the noted molar ratio at 80 °C for 1 ~ 2 h with a magnetic stir bar until a homogeneous liquid was formed. The DESs were then cooled down to room temperature and stored in a desiccator for later use.

### Direct extraction of VFAs with HDESs

In the direct extraction experiment, equal volumes of aqueous solution and extraction solution (HDES) were added to a centrifuge tube and mixed using a laboratory rotator (ATR, Inc., model no. RKVSD) at 60 rpm for 3, 6 or 12 h. Afterwards, the mixtures were settled and separated by centrifugation at 6000 rpm for 2 min. The VFAs concentrations in the aqueous phase before (*c*_*0*_) and after (*c*_*t*_) extraction were measured with an HPLC (Dionex UltiMate 3000) equipped with a refractive index detector and a Biorad Aminex HPX-87 H column. The mobile phase applied was 5 mM H_2_SO_4_ at a flow rate of 0.4 mL/min, and the column temperature was set to 50 °C. All conditions were tested in triplicate.

Since visual inspection after extraction did not reveal measurable changes in aqueous phase volume across all trials, the extraction recovery percentage (ERP = (*c*_*0*_-*c*_*t*_)/*c*_*0*_) of each VFA was calculated to compare the extraction performance, assuming constant aqueous phase volume. Although hydrophobic DESs generally exhibit low water solubility under equilibrium conditions, minor volume changes due to the possible absorption of small amounts of water by the solvent were not experimentally quantified in this study and are noted as a methodological limitation.

### Extraction through a membrane system

The membrane extraction experiment was performed in an omniphobic membrane contactor module with an active membrane area (*A*) of 20.6 cm^2^, as previously detailed and illustrated [27]. The PTFE omniphobic membrane used for testing was purchased from Membrane Solutions, LLC (Part no. OLE100H60, Lot no. 292083136) and had a thickness of 184 ~ 199 μm and a pore size of 1 μm. A batch extraction experiment was conducted by counter-currently flowing a known amount (*V*) of fermentation supernatant (*F-a*) on the feed side and the Thy/DMP (1:2) DES on the draw side of the module at the same flow rate of 70 ml/min using two peristaltic pumps. The *F-a* sample was prepared by filtering the supernatant of a fermentate and adjusting the pH to 3 using hydrochloric acid. The experiment was conducted sequentially with the whole system placed at room temperature (RT, around 25 °C) and 55 °C, respectively.

Samples were taken from the aqueous phase before (*c*_*0*_) and after (*c*_*t*_) a 1-hour operating period (*t*) and analyzed for the VFAs composition using a GC (HP5890, Hewlett Packard, USA) equipped with flame ionization detector and a capillary GC column (NUKOL 15 m × 0.53 mm × 0.50 μm). The column temperature was ramped from 75 °C to 120 °C at a rate of 4 °C/min, and the injection and detector temperature was set to 200 °C and 250 °C, respectively. Nitrogen was used as the carrier gas at a flow rate of 40 ml/min. The average extraction flux (AEF=(*c*_*0*_-*c*_*t*_)×*V*/(*t*×*A*)) of each VFA species was calculated accordingly for each condition.

### Molecular simulation

The association among HDES components and the aimed extracting solutes as well as their interaction with water at the interface were investigated for selected HDESs using molecular dynamics (MD) simulations performed with GROMACS 2020.4 [28] (Table A.1). The molecular model was modified based on previous studies [29, 30]. Briefly, the OPLSAA/M force field [31] was used to describe the HDES-forming components, whose structures are shown in Fig. 1. The force filed parameters were assigned using the Ligpargen web server [32–34] and listed in Table A.2. The TIP3P model was used for water molecules. The short and long ranged non-bonded interactions are calculated using the Lennard-Jones and Coulomb potential [30]. 

To create a HDES system, a total of 300 units, either molecules or formula units for ionic compounds, were inserted randomly into a cubic box using the insert-molecule command of GROMACS. The number of units for each HDES component was based on the noted molar ratio. For example, 100 thymol molecules and 200 DMP molecules were used for the Thy/DMP (1:2) system. The HDES-water system was established based on the corresponding HDES system by directly inserting a certain number of water molecules, whose volume is the same as the equilibrated HDES system at 25 °C. Similarly, the HDES-substrate system was built by inserting 1 formula unit of the aimed extracting solute (i.e., butyric acid or ammonia butyrate) into the corresponding HDES system.

A 6-step simulation process was carried out for each system with the steps described as follows. (1) energy minimization to optimize the geometry of the atoms and remove any too-close contact between atoms. (2) isobaric-isothermal (NPT) ensemble MD simulation (100 ns, *P* = 1 atm, T = 298 K) to relax the simulation box. (3) isometric-isothermal (NVT) ensemble MD simulation (200 ns, *P* = 1 atm, T = 353 K) to accelerate the thermodynamic equilibrium. (4) NVT ensemble MD simulation (200 ns, T = 298–328 K) to achieve the thermodynamic equilibrium of the system. (5) NPT ensemble MD simulation (200 ns, *P* = 1 atm, T = 298–328 K) to allow the bulk phase to reach thermodynamic equilibrium. (6) NPT ensemble MD simulation (500 ns, *P* = 1 atm, T = 298–328 K) data collection with a frequency of 50-ps. The temperatures for step 4 ~ 6 were set to 298–328 K for RT and 55 °C extraction systems, respectively. The integral step was 2 fs in steps 2 ~ 6.

Simulations utilized the periodic boundary conditions. The short and long range nonbonded interactions in the OPLS-AA/M force field are calculated using the Lennard-Jones 12 − 6 and Coulomb potential, respectively (Eq. 1).

1 $$\begin{array}{l}\:E=\sum\:_{i}\:\sum\:_{j<i}\\\left\{\:\frac{1}{4\pi\:{\epsilon\:}_{0}}\:\frac{{q}_{i\:}{q}_{j}{e}^{2}}{{r}_{ij}}+4{\epsilon\:}_{ij}\left[{\left(\frac{{\sigma\:}_{ij}}{{r}_{ij}}\right)}^{12}-\:{\left(\frac{{\sigma\:}_{ij}}{{r}_{ij}}\right)}^{6}\:\right]\:\right\}\end{array}$$

where $\:{r}_{ij}$ is the distance between atom *i* and *j*, $\:{q}_{i\:}{and\:q}_{j}$ are the partial charges of atom *i* and *j*, $\:{\epsilon\:}_{0}\:$is the free space permittivity, $\:{\epsilon\:}_{ij}$ and $\:{\sigma\:}_{ij}$ are energetic and geometric parameters. The Berendsen method [35](steps 2 and 5) and the Parrinello-Rahman [36, 37] method (step 6) were used to control the system pressure in NPT process; the velocity rescaling method was used to control the system temperature; the particle mesh Ewald (PME) [38] sum was used to calculate long-range potentials; and the LINCS algorithm [39] was used to constrain bonds involving hydrogen atoms.

### Statistical analysis

The significance of the results was evaluated using either a t-test or a multi-factor ANOVA, depending on the number of independent factors. The t-test was conducted using Microsoft Excel’s built-in T.TEST function with a two-tailed test. For experiments involving multiple variables, the ANOVA was performed using the dplyr package in R (version 4.2.3), followed by Tukey’s post-hoc test for pairwise mean comparisons. All extraction experiments were conducted in triplicate.

## Results and discussion

### Direct extraction with various HDESs

**Fig. 2:**
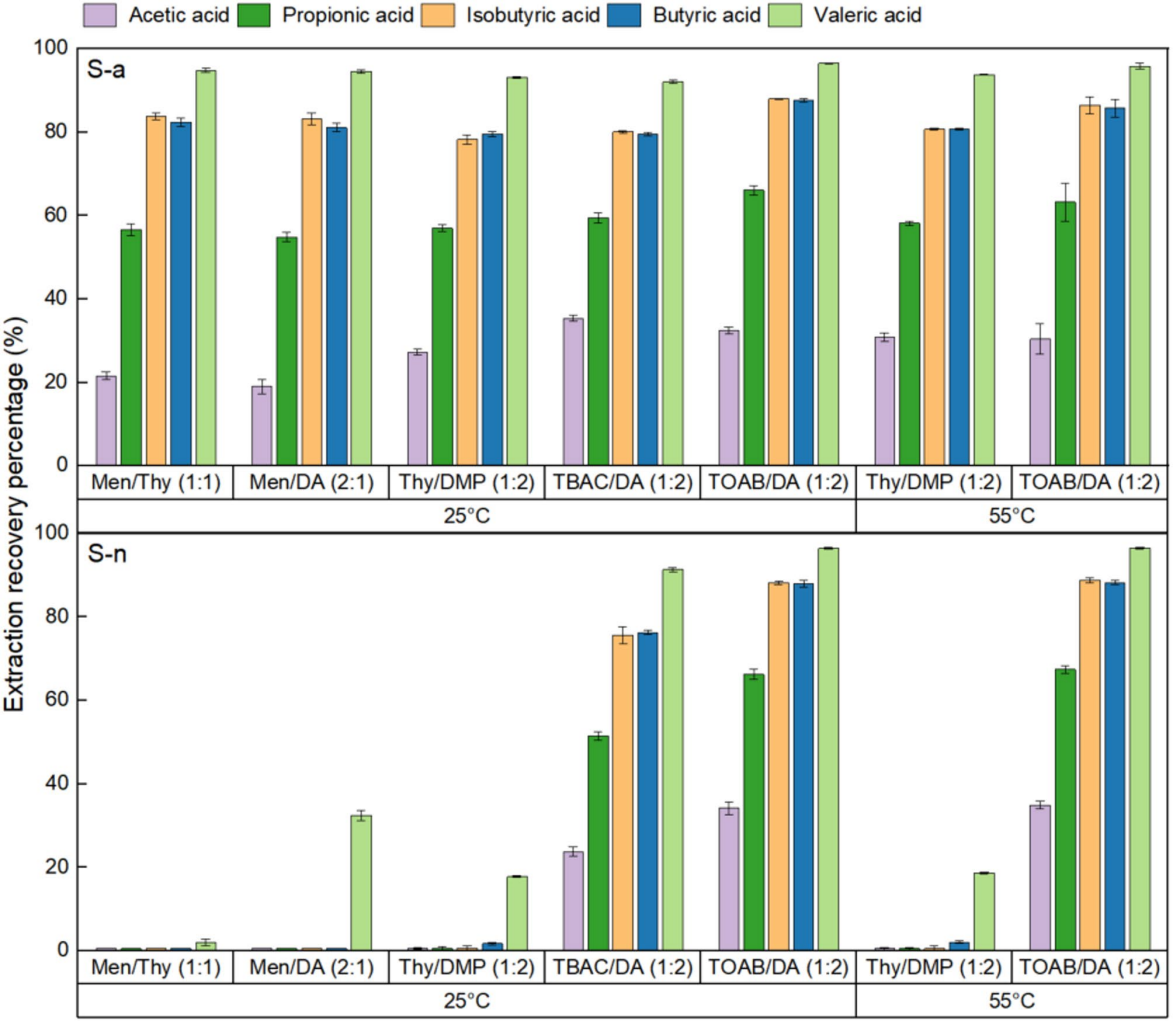
VFAs extraction recovery percentage with HDESs from acidic (S-a) and neutralized (S-n) aqueous solutions under different temperatures

Since no substantial difference in extraction recovery was found after 3, 6 or 12 h in the pre-experimental test (Table A.3), 3 h was considered sufficient to achieve the extraction equilibrium and was applied for all conditions in the direct extraction experiment. This allows for a comparison of extraction efficiency across different HDESs at different extraction temperature and pH (Fig. 2). The ERP value exhibited a positive correlation with the carbon atom number of VFA, with valeric acid showing the highest recovery percentage, while acetic acid having the lowest under each condition. The two-way multi-factor ANOVA of the data collected under 25 °C for acidic (*S-a*) conditions revealed a non-significant impact of the interaction between HDES type and VFA species on the ERP value. These results suggest that ERP values are related to the properties of individual VFAs, while the type of HDES used does not significantly influence this relationship. First, the observed extraction patterns align with established trends showing increased efficiency for acetic, propionic, and butyric acids as their alkyl chain length and corresponding hydrophobicity increases [15, 17]. However, isobutyric acid deviates from this trend, showing comparable extraction efficiency to butyric acid despite its higher aqueous solubility [40, 41], likely due to reduced steric hindrance from its branched structure (17, 24).

#### Effect of temperature on extraction recovery

Temperature could affect the extraction performance by changing the distributions of VFAs between the two phases. The extraction performances of VFAs from thermophilic AF system (55 °C) may differ compared to the RT condition. So, Thy/DMP (1:2) and TOAB/DA (1:2) were chosen as representatives of type V and type III HDESs, respectively, and evaluated for their ability to extract VFAs from acidic and neutralized aqueous phase at 55 °C. The results were then compared to earlier experiments conducted at RT and presented in Fig. 2.

When using either of the two tested HDESs and an aqueous solution with a specific pH level, the recovery for every VFA species remained consistent at 25 °C and 55 °C. The multi-factor ANOVA of the data confirmed that the 30 °C difference between the targeted temperatures did not significantly alter the distributions of the VFA species. Although quantitative data on changes in VFA aqueous solubility from 25 °C to 55 °C are limited in the literature, our MD simulations provide supporting evidence for this observed stability, as detailed in the following section.

#### Effect of pH on extraction recovery

The conventional process for recycling/extracting VFAs from fermentate involves first adjusting the effluent to a low pH, resulting in additional costs on acidifying reagent and more complicated downstream processes [6]. Ideally, if VFAs could be removed from the effluent without altering its physical-chemical characteristics, a truly continuous VFA producing system becomes possible since VFAs (product inhibition) could be removed from the effluent in-situ. To evaluate the effect of pH on extraction performance, direct extraction experiments were conducted at room temperature comparing a 10 mM VFAs solution at acidic pH of 3 (*S-a*) and a close neutral condition with pH adjusted to 6 (*S-n*). A 10% ammonia solution was employed for pH adjustment to simulate the alkaline composition in real fermentate. As shown in Fig. 2, under S-a condition, all types of HDESs showed considerable extraction recovery, particularly for 4 C- and 5 C- VFAs, which were over 80% and 92%, respectively. However, upon neutralizing the aqueous solution (*S-n*), only TBAC/DA (1:2) and TOAB/DA (1:2) maintained relatively high ERPs like *S-a*, while all other types of HDES showed a dramatic decrease in ERPs. A three-way multi-factor ANOVA was conducted on the dataset collected under 25 °C. The results indicated a significant influence of pH on the ERP value across various VFA species. Specifically, the type V HDESs including Men/Thy (1:1), Men/DA (2:1), and Thy/DMP (1:2) exhibited a pronounced sensitivity to pH difference. While the type III HDESs TBAC/DA (1:2) and TOAB/DA (1:2), demonstrated no statistically significant pH-dependent effects on their respective ERP values. The reduced extractability of type V HDESs at higher pH may come from the enhanced dissociation of VFA molecules into their conjugate salts. The studied VFAs exhibit pKa values spanning 4.60–4.87 [42, 43], for which the Henderson-Hasselbalch equation predicts 93%-96% dissociation into conjugate base forms at pH 6. These ionized species generally possess stronger polarity and are more likely to stay in polar solvents such as water than in HDESs. The ionic component of TBAC or TOAB in type III HDESs may have played an important role in facilitating the extraction of VFA at neutral pH for the TBAC/DA (1:2) and TOAB/DA (1:2) HDESs. A more in-depth investigation was conducted by MD simulation and discussed in next section.

### Molecular simulation

MD simulations were conducted to investigate the altered performance of different HDESs in extracting VFAs under varying conditions. The Thy/DMP (1:2) and TOAB/DA (1:2) systems, representing type V molecule-compound-based and type III ionic-compound-based HDESs, respectively, were selected for the simulation. The interface between the HDES and water phases was studied by simulating a system that initially contained both HDES-forming compounds and a comparable volume of water molecules. Since increasing pH could enhance the dissociation of VFAs and lead to more carboxylate salts in the aqueous phase, we assume that the main difference between the acidic (*S-a*) and neutralized (*S-n*) solutions is the change in the extracting substrates from VFA molecules to the corresponding carboxylate salts. Given this assumption, to simulate the extraction process in *S-a* and *S-n* conditions, one butyric acid (BA) molecule or one formula unit of ammonium butyrate (But^−^) was added as the extracting substrate to the HDES system, respectively. The butyric compound was chosen as a representative not only because it was easily extracted to the studied HDESs in the previous section, but also because of its potential as a platform chemical and its abundance in fermentation effluents. The pure water systems were also studied for comparison which were inserted with one butyric acid molecule or one formula unit of ammonium butyrate. The systems were stabilized at 25 °C and 55 °C as needed to explain the effect of temperature on the extraction performance.

#### Effect of temperature on interactions among HDES and VFA compounds

**Fig. 3:**
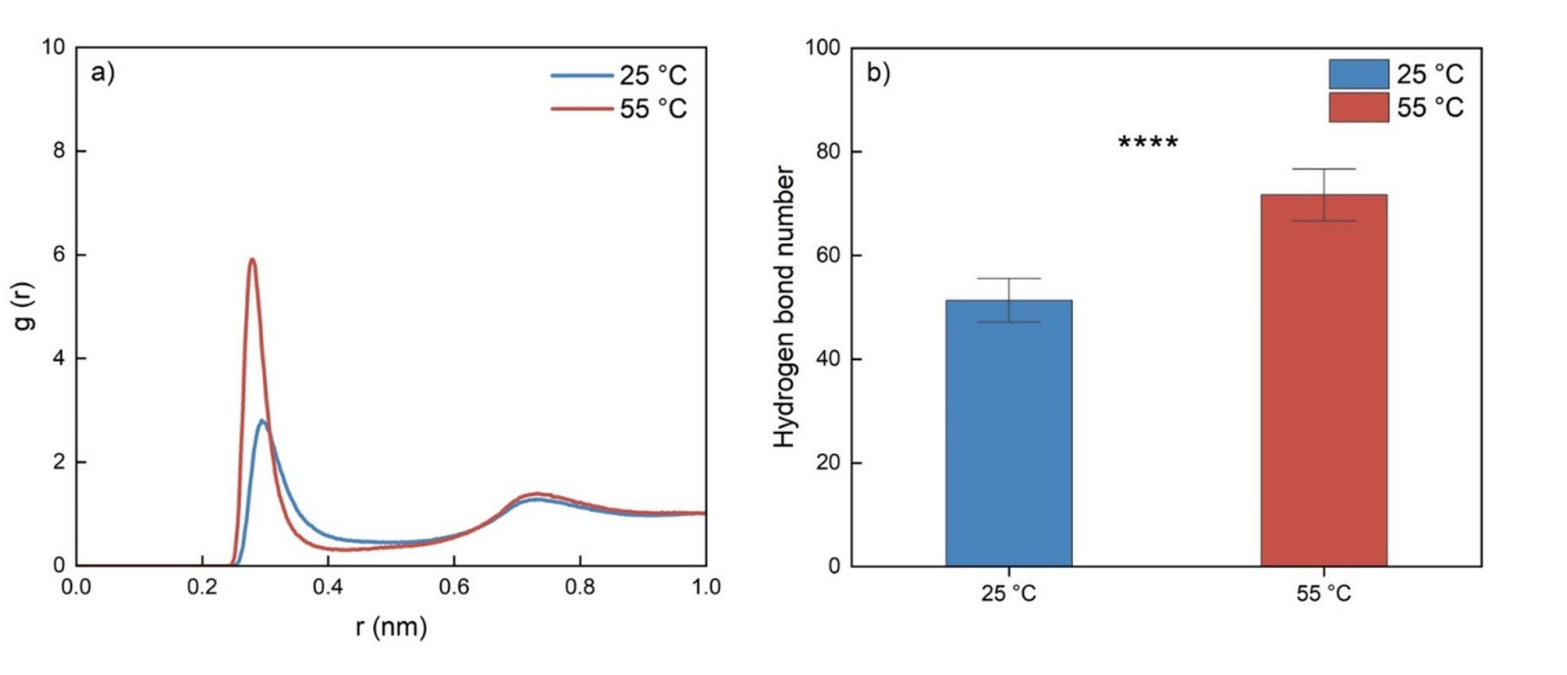
Effect of temperature on the interaction between the HDES-forming components in the Thy/DMP (1:2) system, represented by (**a**) radial distribution functions (RDFs, g(r)) between the oxygen atoms of Thy__O0A_ and DMP__O0A_; and (**b**) hydrogen bond number between thymol (100 molecules) and DMP (200 molecules) under 25 °C and 55 °C (**** indicates *p* ≤ 0.0001)

**Fig. 4:**
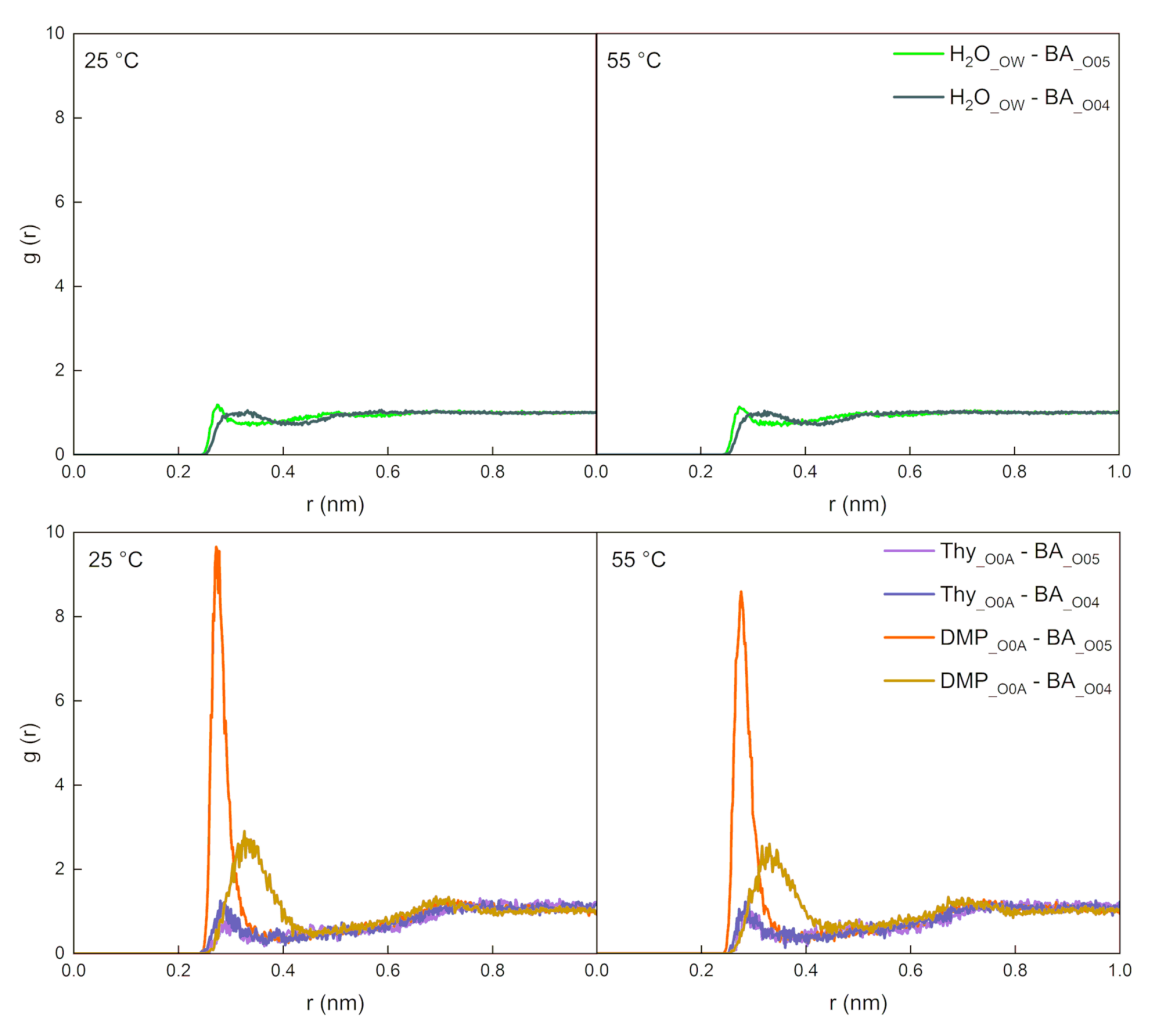
RDFs (g(r)) between the oxygen atoms in butyric acid and solvents (water or Thy/DMP (1:2) HDES) under 25 °C and 55 °C

The O-O radial distribution functions (RDFs, g(r)) were analyzed to characterize molecular associations in the HDES systems [30] and between the solvent and BA substrate at 25 °C and 55 °C (Figs. 3 and 4). The RDF quantifies the probability density of locating a specific atomic or molecular site at a distance r relative to a reference particle, normalized by the bulk density. In these figures, the first peak at ~ 0.3 nm typically corresponds to direct association between the two oxygen atoms, while subsequent peaks at larger distances arise from structural arrangements influenced by neighboring atoms in the solvent matrix. Higher g(r) values at these peaks indicate stronger associations [25].

According to our former research, among the 3 oxygen atoms in DMP, DMP__O0A_ has the strongest association with the functional Thy__O0A_ atom of thymol and contributed the most to the formation of hydrogen bonds within the Thy/DMP (1:1) HDES [25]. So, the higher RDFs peak observed between DMP__O0A_ and Thy__O0A_ at 55 °C compared to 25 °C, as shown Fig. 3a, suggests that the interaction between DMP and Thy molecules in the HDES system is stronger at higher temperature. The higher average number of hydrogen bond at 55 °C is also in agreement with this conclusion (Fig. 3b).

The results in Fig. 4 explain the satisfactory ERP of Thy/DMP (1:2) towards butyric acid at *S-a* condition and demonstrate that temperature has a negligible effect on the extraction performance. For aqueous and HDES systems, the predominant peaks within 0.3 nm are of BA__O05_-H_2_O__OW_ interaction and BA__O05_-DMP__O0A_, respectively. So, the hydroxyl group in the BA molecule contributed to the dissolving of BA into both systems. And in the Thy/DMP (1:2) system, the hydroxyl group in DMP is crucial to stabilize BA molecules in such HDES systems. The smaller peak of BA__O05_-H_2_O__OW_ compared to BA__O05_-DMP__O0A_ indicated a tendency for BA to be drawn from aqueous phase into the HDES system. Furthermore, it is worth noting that the RDFs results for these functioning peaks show no significant difference when the temperature setting changed from 25 °C to 55 °C, indicating that the small temperature change of 30 °C has little effect on the interaction between the substrate and solvents. This finding provides additional evidence and supports the experimental results, where the ERP of VFAs remained unchanged when the surrounding temperature increased from 25 °C to 55 °C.

#### Density distribution of HDES components and water molecules in HDES-Water systems

**Fig. 5:**
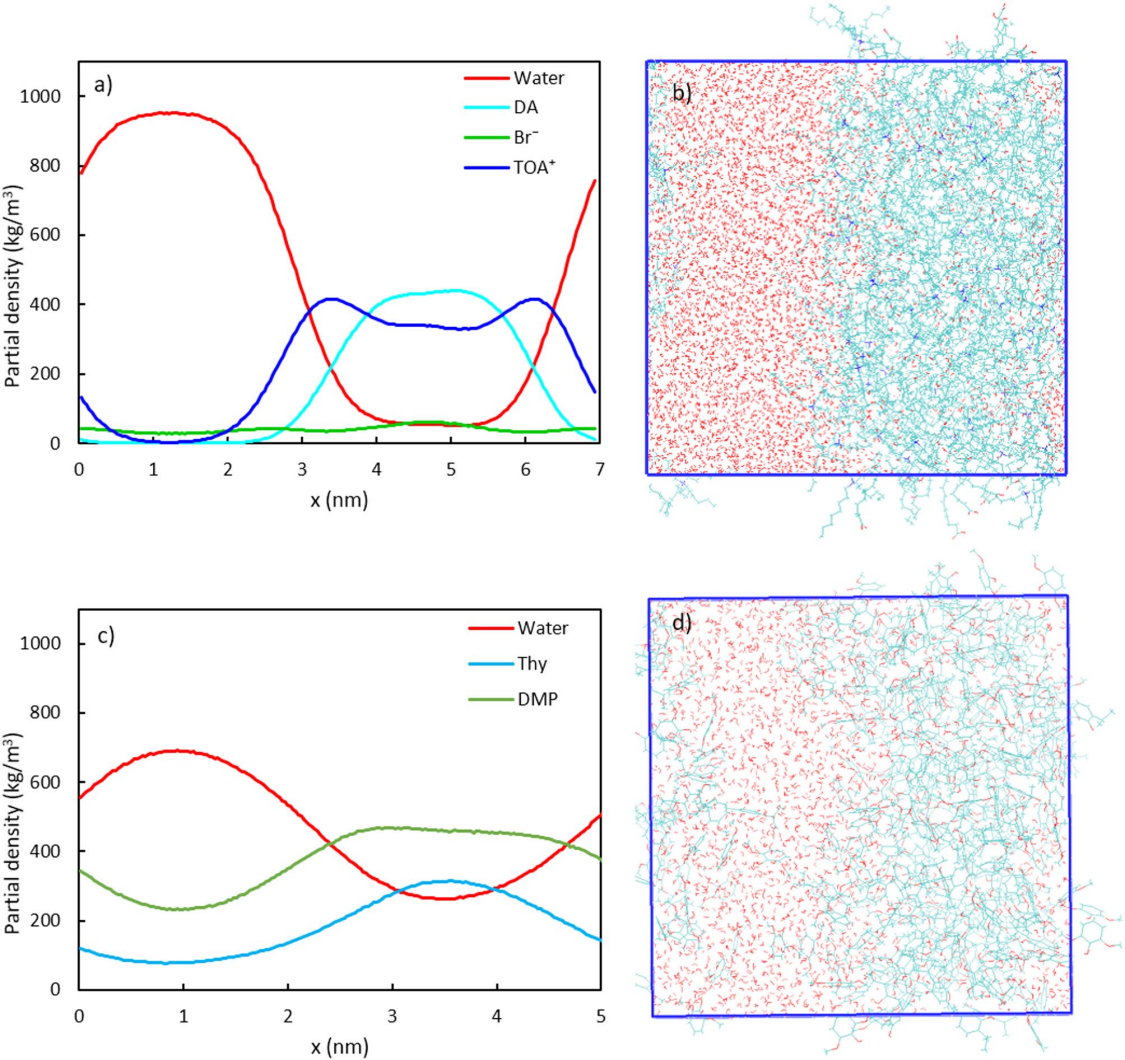
Simulation results of HDES-water systems: (**a**) and **c**) are density profiles of systems TOAB/DA (1:2) and Thy/DMP (1:2), respectively; (**b**) and **d**) are the snapshot of the simulation boxes for system TOAB/DA (1:2) and system Thy/DMP (1:2), respectively. The molecules are shown in the wireframe model (C: cyan: O: red, N: blue, and H: white)

In the direct extraction experiments, a distinct liquid-liquid interface was found between the aqueous solution and any of the tested HDESs. The biphasic mixtures containing water and the representative TOAB/DA (1:2) and Thy/DMP (1:2) HDESs were simulated at 55 °C, and the results are presented in Fig. 5. The snapshots of the simulation boxes provide us with an intuitive understanding of the separation of HDESs components from water. The TOAB/DA (1:2) – water system exhibits a clear interface, while the Thy/DMP (1:2) – water system displays a somewhat blurred interface, as observed from the simulation snapshots. The partial density profiles confirm these observations, as a well-defined liquid–liquid interfacial region of around 2.6 nm can be read for the TOAB/DA (1:2) – water system based on the distance between bulk HDES and water phases, while the partial density of water in the Thy/DMP (1:2) – water system does not reach a plateau throughout the simulation box. This means that, for the later system, the three solvent components (i.e., thymol, DMP and water) all exist in both aqueous and hydrophobic phases but with different proportions. This situation differs from previous simulation results for the same system at 25 °C, where HDES components did not dissolve in the bulk aqueous phase, as indicated by the corresponding density profile curves touching zero in the bulk water region [44]. This suggests that the increase in temperature could have enhanced the mixing of the two phases, particularly the absorption of HDESs components into the aqueous phase.

The size of the stabilized pure Thy/DMP (1:2) box was smaller than that of the TOAB/DA (1:2) system because of the relatively smaller size of individual Thy and DMP molecules. Following the rule to insert water molecules with a comparable volume to the HDES phase, 2165 water molecules were used in the Thy/DMP (1:2)-water system, which was fewer than that used in the TOAB/DA (1:2) system (5322 molecules). As a result, the Thy/DMP (1:2)-water box was slightly smaller (side length 5.1 nm). To exclude the effect of constrained space on the separation of water and HDES components in this smaller box, a similar system containing increased number of water molecules (4300) was built and stabilized to a bigger box (side length 6 nm). However, no plateau was found for the water curve either, substantiating that the biphasic Thy/DMP (1:2) – water system was more thoroughly mixed at 55 °C. Interestingly, increasing the number of water molecules resulted in a higher water partial density in the bulk HDES region, but the distribution of HDES components remained the same. This finding is consistent with previous research by [29] that suggests water molecules can more easily dissolve in the HDES phase than vice versa. The miscibility of water and HDES shall come from the stronger HDES-water interaction, and the water molecules that enter the HDES phase may alter the properties of the Thy/DMP (1:2) HDES by adjusting their hydrogen bonds.

The TOA^+^ peak observed at the interface in the TOAB/DA (1:2) – water system indicates a preferential accumulation of the tetraoctylammonium cations at the interface, and this may create an additional attraction force upon the butyrate anions and assist their extraction into the TOAB/DA (1:2) phase at neutral pH. Furthermore, despite the well-separated aqueous and HDES phases, Br^−^ anion was found to spread throughout the entire system. This phenomenon supports the possibility of ion exchange during the extraction, which could also play an important role in facilitating the transfer of butyrate anions [18]. The ion exchange mechanism, however, involves irreversible interaction where recovery of extracted VFA conjugate salts requires reintroducing the original leached anions (e.g., Br⁻ or Cl⁻) displaced during extraction [18]. While a patent has demonstrated successful HDES regeneration through hydrochloric acid supplementation for metal recovery systems [45], this approach remains unvalidated for VFA extraction scenarios. Further investigation must address whether similar methods can restore HDES functionality for VFA recovery, alongside economic viability assessments of acid consumption and solvent reuse cycles to determine suitability for its application with AF effluents.

#### Effect of pH on the HDES-substrate interactions

**Fig. 6:**
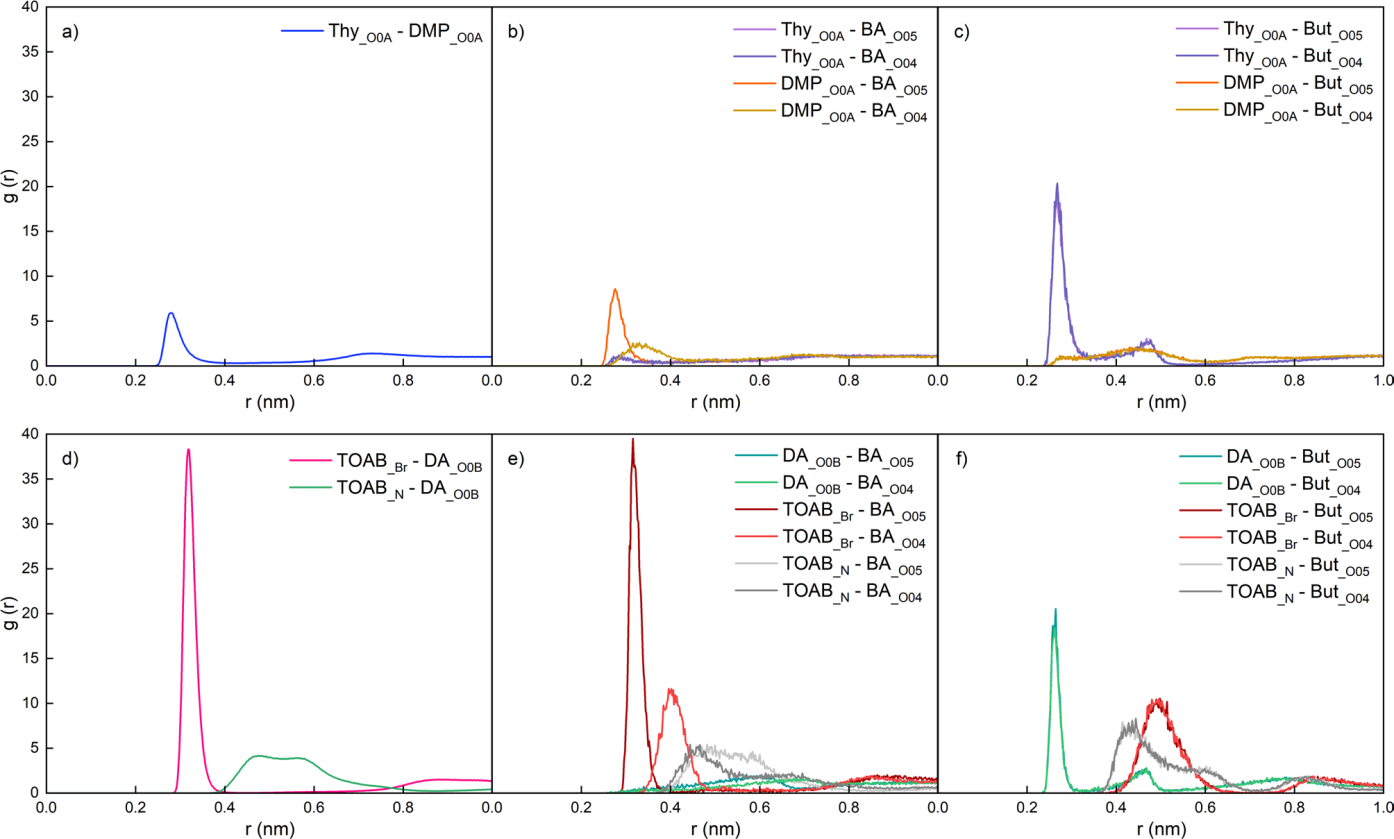
Molecular dynamic simulation results for HDES-substrates systems at 55 °C represented by RDFs (g(r)) between the functional atoms of HDES-forming molecules or with solute formula units. Systems analyzed include (**a**) Thy/DMP (1:2), (**b**) BA in Thy/DMP (1:2), (**c**) But⁻ in Thy/DMP (1:2), (**d**) TOAB/DA (1:2), (**e**) BA in TOAB/DA (1:2) and (**f**) But⁻ in TOAB/DA (1:2)

The results of RDF between representative atoms of HDES components and substrates at 55 °C are summarized in Fig. 6, which are presented using a uniform axis range. For the Thy/DMP (1:2) system, the DMP__O0A_ atom was chosen to typify the oxygen atoms in DMP and was analyzed about its RDF with the thymol oxygen (Thy_ _O0A_) (Fig. 6a). In this interaction, thymol was found to primarily act as a hydrogen bond donor (HBD) and DMP as the hydrogen bond acceptor (HBA), as demonstrated by MD simulations in a previous study [25] showing a higher proportion of ThyOH-DMP__O0A_ than Thy__O0A_-DMPOH in the hydrogen bonds quantity analysis. In the HDES systems containing BA or But^−^, the DMP__O0A_ and Thy__O0A_ atoms were also used to analyze the RDFs with the oxygen atoms in the solutes (Fig. 6b, c). In the BA-containing system, the interaction of DMP__O0A_-BA__O05_ contributes the most to the affinity of BA molecules to this HDES species. On the contrary, for the But^−^-containing system, Thy__O0A_ is the major contributor and its interaction with the two oxygen atoms are of the same strength, demonstrated by the identical peaks of Thy__O0A_-But^−^__O05_ and Thy__O0A_-But^−^__O04_. Considering that carboxylic acids are frequently used as the HBDs to formulate HDESs with various types of HBA like menthol and quaternary ammonium salts [20, 46], in the BA-containing system, it is possible that BA could also form hydrogen bonds with the HBA of the HDES (i.e., DMP). The similar heights of Thy__O0A_-DMP__O0A_ (Fig. 6a) and BA__O05_-DMP__O0A_ (Fig. 6b) peaks suggest a comparable hydrogen bonding strength between BA-DMP and Thy-DMP molecules, which should have ensured the stabilization of BA molecules in this HDES system. However, when a But^−^ anion was inserted, thymol instead of DMP plays the role to attract the solute. Moreover, the interaction between Thy and But^−^ is much stronger than the hydrogen bonding of Thy-DMP, as denoted by the higher Thy-But^−^ peaks in Fig. 6c compared to 5a. This strong interaction can be attributed to the ion-dipole interactions between the But⁻ anion and thymol molecules. In this case, the electron resonance in the carboxylate anion negatively charges the two oxygens (i.e., But^−^__O05_ and But^−^__O04_) equally and the delocalized electron attracts the positive end of the polar thymol molecule, resulting in the identical large Thy__O0A_-But^−^__O05_ and Thy__O0A_-But^−^__O04_ RDF peaks observed in Fig. 6c. The strong ion-dipole interaction between the But^−^ anion and thymol molecule may have weakened the Thy-DMP interaction within the HDES, leading to the inability to extract butyrate from the aqueous phase at neutral pH (47). This suggests that, But^−^ anion tends to compete for thymol against DMP, thus destabilizing the HDES system near the anion and eventually disrupting the extraction process.

This theory could also be applied to explain the similar extraction performance observed in other type V HDESs investigated in this study, where menthol was demonstrated to act as HBA, while thymol or DA as HBD in the Men/Thy (1:1) and Men/DA (1:2) HDESs, respectively [21, 30]. When the aimed solutes are the VFA molecules (i.e., at low pH), they could act as alternative HBDs in the HDES and form H-bonds with menthol. As a special type of dipole-dipole interaction, all these neutral H-bonds formed between type V HDES components or with VFA molecules are comparable in interaction strength [47, 48] and are generally weaker than the ion-dipole interaction, which is recognized between the HBD of HDESs (i.e., thymol or DA) and the VFA conjugate bases (e.g., But^−^) in Fig. 6c, f. In this case, at neutral pH and the VFA conjugate base anions are the dominant solutes, their strong ion-dipole interaction with the HBD of HDES could weaken the surrounding portion of HDES system and consequently hinder the extraction [49].

As for the TOAB/DA (1:2) system, the preference of different solutes to the two components of HDES follows the same rule, where the BA molecule as an HBD (i.e., DA) analogue has stronger affinity to the HBA (i.e. TOAB), while the But^−^ anion associates with the HBD of the HDES (Fig. 6d-f) [50]. In contrast, the prominently high peak in Fig. 6d indicates that the interaction within the HDES components should be inducted to anionic hydrogen bonding [51], where the negative charge on the HBA fragment (i.e., Br^−^) is apt to add extra interaction energy to the hydrogen bond [52] with the neutral HBD molecule (i.e., DA). Therefore, the strong ion-dipole interaction between the But^−^ anion and DA molecule, as denoted by the sum of the two DA__O0B_-But^−^__O_ peaks in Fig. 6f, become comparable to that within the type III HDES (Fig. 6d). As a result, it is not able to destroy the HDES system and the extraction process in the pH-neutral system was enabled.

### Extraction from synthetic mixture and real fermentate

Based on the direct extraction experiment and MD simulation results discussed above, it was demonstrated that all the HDESs studied were effective extractants for VFAs from a synthetic aqueous solution. Furthermore, the type III HDESs were found to be capable of extracting the conjugate base of VFAs from a neutral pH system, which is in the same pH range as that of a real AD fermentate. However, unlike the synthetic solution that simply contains known VFA species with fixed concentration (10 mM), real fermentate is much more complex and contains various other AD constituents such as ammonia, phosphorus compound, furans, and extractives from feedstock, among others. Additionally, the VFA profile in real fermentate varies and is typically higher in acetic, propionic, and butyric acids, but lower in isobutyric and valeric acids.

**Fig. 7:**
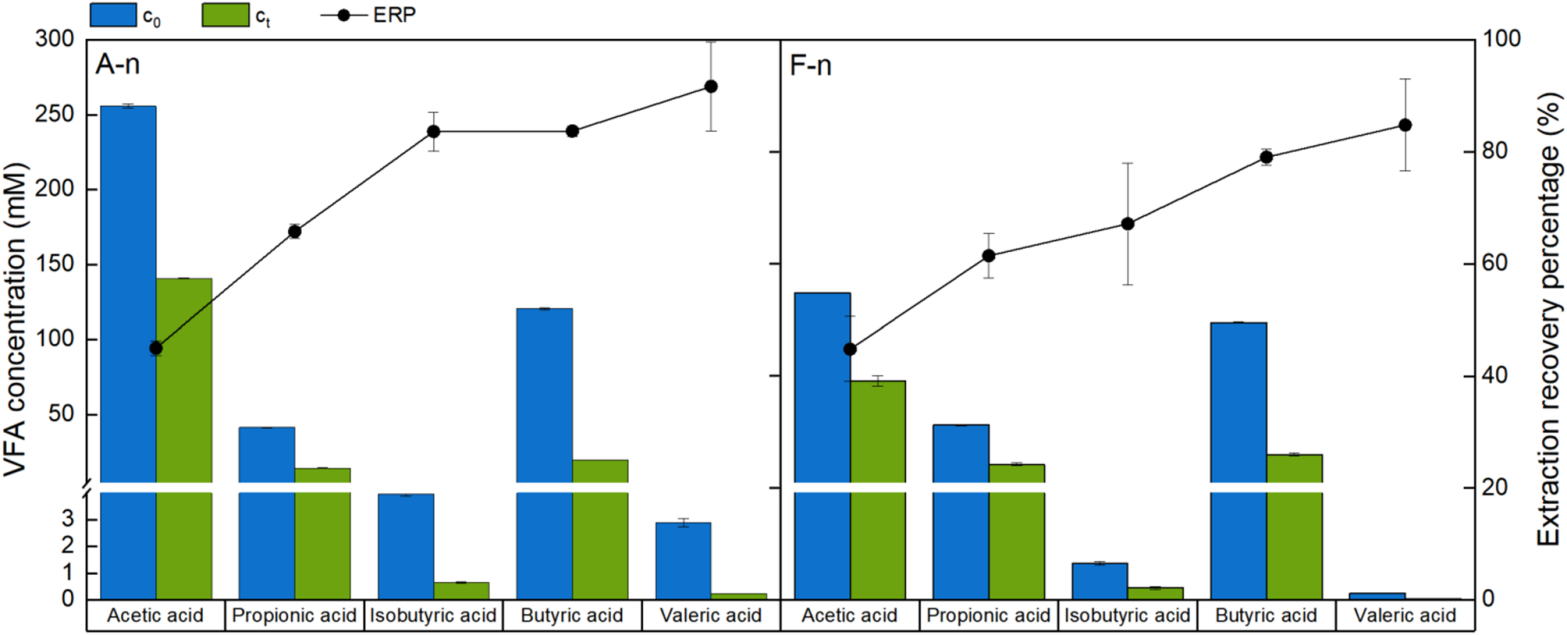
VFA concentrations before and after extraction (bar plot) and extraction recovery percentages (connected scatterplot) for the synthetic mixture (left) and filtered real fermentate (right), extractant: TBAC/DA (1:2), t = 3 h

To examine the impact of these differences, an artificial neutralized aqueous condition (*A-n*) with referenceable VFA profile was tested using TBAC/DA (1:2) at RT, and a real filtered supernatant of the actual fermentate (*F-n*) was studied subsequently under the same condition. The concentration before and after extraction (*c*_*0*_ and *c*_*t*_, respectively) and the ERP of each VFA species under both conditions are presented in Fig. 7, with the corresponding data provided in Table A.4. Comparing the results with that in Fig. 2, it is found that the alteration in the VFA profile did not affect the ERP trends for different VFA species. For instance, TBAC/DA (1:2) was still capable of extracting neutralized VFAs from either the artificial or real fermentate despite their differing VFAs contents. This scenario can be attributed to the fact that the concentrations of volatile fatty acids (VFA) are generally very low. Taking acetic acid as an example, the increase in concentration from 10 mM to 250 mM corresponds to a volume percentage change of only between 0.6% and 1.4% in the aqueous phase. Given these minimal changes in concentration, it is expected that the equilibrium would not be substantially affected. Although a specific ternary phase diagram is unavailable to substantiate this claim, the close proximity of the concentration points, as expected from general knowledge, implies a negligible influence on the equilibrium.

The higher concentration of certain VFA types, including acetic, isobutyric and valeric acids, in the *A-n* condition compared to the *F-n* condition is because that the *A-n* solution was prepared to contain the highest obtainable concentration for each VFA species during a batch acidogenic fermentation process and does not necessarily reflect the specific VFA profile of a particular fermentate. Given that the *A-n* solution was adjusted using an ammonia solution to match the pH range of the actual fermentate (*F-n*, pH 6), the primary difference between the two aqueous samples was the presence of soluble impurities in the real fermentate. Comparing the ERP values of the two conditions, no apparent difference was found for all VFA species, demonstrating a negligible effect of such soluble impurities in the real fermentate on the extraction performance.

### Extraction using a membrane system

Membrane extraction is a sustainable liquid-liquid extraction technique that has the potential to reduce extractant requirements and minimize emulsion-related contamination risks, particularly when processing complex substrates such as AD fermentate. Theoretically, such technique supports the long-term goal to build a truly continuous VFA producing and extracting system by recycling the two phases separately. Through real-time distillation, VFAs can be efficiently extracted while the extractant is regenerated and reused within the process, thereby minimizing the total extractant volume required for sustained operation [27]. To evaluate the feasibility of this process, a membrane assisted extraction experiment was conducted using a membrane contactor module equipped with an omniphobic membrane with a real fermentation effluent adjusted to pH 3 (denoted as *F-a*) on the feed side and the relatively cost-effective Thy/DMP (1:2) HDES on the draw side [27]. The omniphobic membrane, characterized by ultralow surface energy and re-entrant structures, effectively repels liquids and exclusively permits vapor-phase compounds [53–56], such as undissociated VFAs, to pass through, thereby preventing membrane wetting by liquids from both the feed and draw sides. So, for this experiment, the fermentation effluent was adjusted to pH 3 to ensure that the majority of VFAs became undissociated (volatile) [27], and enable their passage through the omniphobic membrane as VFA vapor, driven by the partial pressure differences of undissociated VFA molecules between the feed and draw solutions. Upon reaching the draw side, they were effectively captured by the HDES due to the hydrophobic affinity.

Table 2 presents a comparison of the AEF values for VFAs obtained in this study with those reported for a PTFE-TOA membrane system employing 0.5 N NaOH on the permeate side, a configuration previously demonstrated to be effective for VFA recovery [12]. The omniphobic membrane contactor module used in this study yielded AEF values for all VFA species that were slightly lower, yet still comparable to those achieved with the established PTFE-TOA system. The increased thickness of the omniphobic membrane applied here may have contributed to the decrease of AEF in general. Given that the VFA partial pressure gradient between the two sides of the membrane is among the driving forces for effective extraction, the different concentrations of the various VFA species could also impact their specific extraction rates. As a result, the AEF values of the most abundant VFA species (i.e., acetic, and butyric acids) in the AD fermentate are much higher than the others.

**Table 2:** Effect of temperature on the average extraction flux (g/m^2^·h) of VFAs in the membrane contractor modules under different conditions

Temperature	Acetic acid	Propionic acid	Isobutyric acid	Butyric acid	Valeric acid	Membrane	Extractant	Reference
RT	4.1	0.0	0.0	5.1	4.0	Omniphobic membrane	Thy/DMP (1:2)	Current study
55 °C	8.2	3.7	2.1	9.2	2.4	Omniphobic membrane	Thy/DMP (1:2)	Current study
21 °C	5	6	-	7	8	PTFE-TOA membrane	0.2 N NaOH	Ref [12].
38 °C	9	11	-	12	13	PTFE-TOA membrane	0.2 N NaOH	Ref [12].

Moreover, it is proven that the extracting rates for VFAs were generally higher at 55 °C than RT, which shall be attributed to the faster molecular motion under higher temperature. This suggests the appropriateness of this membrane extraction system to the thermophilic AD fermentate. The only exception against this observation was valeric acid, which showed a slight decrease of AEF value at increased processing temperature. Despite the possibilities of operating errors, this phenomenon may be due to the increased miscibility between water and Thy/DMP HDES at 55 °C, as previously discussed regarding the MD results on density distribution. Specifically, trace amounts of water vapor that may have transferred to the draw side at the elevated temperature may have interacted more strongly with the HDES components, thereby reducing their capability to form hydrogen bonds with VFAs and diminishing their affinity for VFA extraction. Valeric acid’s longer carbon chain renders it a more favored HBD by promoting solvent structuring [48], making this effect particularly pronounced and likely outweighing the benefits of accelerated molecular motion, ultimately reducing its AEF at the elevated temperature.

In short, our study validates the effectiveness of the membrane extraction technique in assisting continuous VFA extraction using a HDES (i.e., Thy/DMP (1:2)), with a comparable flux to previous research. The technique is highly suitable for thermophilic AD fermentation, where the higher temperature facilitates the rapid transfer of VFA molecules, reducing the side effect of the inherently high thickness of the omniphobic membrane, which is known to reduce flux [27]. However, further studies are needed to identify more suitable HDES extractants and demonstrate the system at scale.

## Conclusions

In-situ separation and recovery of VFAs from acidogenic fermentate is essential for an efficient continuous VFA producing system. Considering the specific properties in the effluent, the promising HDESs were examined to extract VFA from aqueous solutions at varying pH and temperature. Type III HDESs were shown to effectively extract VFA from neutralized aqueous solution, indicating their potential for VFA recovery from AF effluent without the need for acidification. This idea was supported by further experiments using real fermentate from a thermophilic AF system, where a type III HDES achieved comparable ERPs to those obtained with neutralized artificial solution. Compared to room temperature, experiments conducted at thermophilic condition (55 °C) did not lead to significant increase of VFA distribution in the extractant, but the higher temperature may promote more through mixing between the two phases and enhanced the cross-membrane mass transfer. This limited effect of temperature was further supported by MD simulations, which showed minimal variation in substrate-solvent affinity across the tested range (25 °C and 55 °C). Additionally, the simulations explained the reduced extraction efficiency of type V HDES compared to type III HDES at pH 6, attributing it to the strong ion-dipole interactions between VFA conjugate bases and HBDs that may have destabilized the type V HDES. A membrane-assisted extraction experiment using a type V HDES with acidified real fermentate achieved a VFA flux comparable to previous work. This study demonstrates the feasibility of using the membrane system to extract VFAs and its suitability for integration with the thermophilic AF process.

## Supplementary Information

Below is the link to the electronic supplementary material.

Supplementary Material 1

## Data Availability

No datasets were generated or analysed during the current study.

## Abbreviations

VFAs: Volatile fatty acids
HDES: Hydrophobic deep eutectic solvents
ERP: Extraction recovery percentages
AD: Anaerobic digestion
AF: Acidogenic fermentation
TOA: Trioctylamine
PTFE: Polytetrafluoroethylene
Men: Menthol
Thy: Thymol
DA: Decanoic acid
DMP: 2,6-dimethoxyphenol
TBAC: Tetrabutylammonium chloride
TOAB: Tetraoctylammonium bromide
AEF: Average extraction flux
MD: Molecular dynamics
ANOVA: Analysis of Variance
RDF: Radial distribution function
HBD: Hydrogen bond donor
HBA: Hydrogen bond acceptor

## References

[1] Ramos-Suarez M, Zhang Y, Outram V. Current perspectives on acidogenic fermentation to produce volatile fatty acids from waste. Reviews Environ Sci Bio/Technology. 2021;20(2):439–78.

[2] Wainaina S, Lukitawesa, Kumar Awasthi M, Taherzadeh MJ. Bioengineering of anaerobic digestion for volatile fatty acids, hydrogen or methane production: a critical review. Bioengineered. 2019;10(1):437–58. 31570035 10.1080/21655979.2019.1673937 PMC6802927

[3] Lukitawesa, Patinvoh RJ, Millati R, Sarvari-Horvath I, Taherzadeh MJ. Factors influencing volatile fatty acids production from food wastes via anaerobic digestion. Bioengineered. 2020;11(1):39–52. 31880192 10.1080/21655979.2019.1703544 PMC7571609

[4] Sarkar O, Rova U, Christakopoulos P, Matsakas L. Influence of initial uncontrolled pH on acidogenic fermentation of brewery spent grains to biohydrogen and volatile fatty acids production: optimization and scale-up. Bioresour Technol. 2021;319:124233. 33254458 10.1016/j.biortech.2020.124233

[5] Atasoy M, Owusu-Agyeman I, Plaza E, Cetecioglu Z. Bio-based volatile fatty acid production and recovery from waste streams: current status and future challenges. Bioresour Technol. 2018;268:773–86. 30030049 10.1016/j.biortech.2018.07.042

[6] Begum S, Arelli V, Anupoju GR, Sridhar S, Bhargava SK, Eshtiaghi N. Optimization of feed and extractant concentration for the liquid–liquid extraction of volatile fatty acids from synthetic solution and landfill leachate. J Industrial Eng Chem. 2020;90:190–202.

[7] Playne M, Smith B. Toxicity of organic extraction reagents to anaerobic bacteria. Biotechnol Bioeng. 1983;25(5):1251–65. 18548758 10.1002/bit.260250508

[8] Bóna Á, Bakonyi P, Galambos I, Bélafi-Bakó K, Nemestóthy N. Separation of volatile fatty acids from model anaerobic effluents using various membrane technologies. Membranes. 2020;10(10):252. 32987682 10.3390/membranes10100252 PMC7598613

[9] Gryta M, Barancewicz M. Separation of volatile compounds from fermentation broth by membrane distillation. Green Sci. 2011;13(3):56–60.

[10] Zhu X, Leininger A, Jassby D, Tsesmetzis N, Ren ZJ. Will membranes break barriers on volatile fatty acid recovery from anaerobic digestion?? ACS ES&T Eng. 2020;1(1):141–53.

[11] Bitas D, Samanidou V, Kabir A, Lucena R, Cárdenas S. Membrane sorptive phases. Analytical sample Preparation with Nano-and other High-Performance materials. Elsevier; 2021. pp. 199–228.

[12] Aydin S, Yesil H, Tugtas AE. Recovery of mixed volatile fatty acids from anaerobically fermented organic wastes by vapor permeation membrane contactors. Bioresour Technol. 2018;250:548–55. 29197778 10.1016/j.biortech.2017.11.061

[13] Wardell JM, King CJ. Solvent equilibriums for extraction of carboxylic acids from water. J Chem Eng Data. 1978;23(2):144–8.

[14] Inyang V, Lokhat D. Butyric acid reactive extraction using trioctylamine in 1-decanol: response surface methodology parametric optimization technique. Arab J Sci Eng. 2021;46(7):6567–77. 10.1038/s41598-020-59273-z PMC7016146 32051536

[15] van den Bruinhorst A, Raes S, Maesara SA, Kroon MC, Esteves ACC, Meuldijk J. Hydrophobic eutectic mixtures as volatile fatty acid extractants. Sep Purif Technol. 2019;216:147–57.

[16] Yang ST, White SA, Hsu ST. Extraction of carboxylic acids with tertiary and quaternary amines: effect of pH. Industrial Eng Chem Res. 1991;30(6):1335–42.

[17] van Osch DJ, Zubeir LF, van den Bruinhorst A, Rocha MA, Kroon MC. Hydrophobic deep eutectic solvents as water-immiscible extractants. Green Chem. 2015;17(9):4518–21.

[18] Reyhanitash E, Zaalberg B, Kersten SR, Schuur B. Extraction of volatile fatty acids from fermented wastewater. Sep Purif Technol. 2016;161:61–8.

[19] Tonova K, Zhivkova S, Lazarova M, Mustafa AJRC, Engineering. Extraction by ionic liquids for the case of detoxification of lignocellulosic hydrolysates. Reaction Chem Eng. 2024;9(10):2610–22.

[20] Florindo C, Branco LC, Marrucho IM. Development of hydrophobic deep eutectic solvents for extraction of pesticides from aqueous environments. Fluid Phase Equilibria. 2017;448:135–42.

[21] Martins MA, Crespo EA, Pontes PV, Silva LP, Bülow M, Maximo GJ, et al. Tunable hydrophobic eutectic solvents based on terpenes and Monocarboxylic acids. ACS Sustainable Chem Eng. 2018;6(7):8836–46.

[22] Kamar MN, Paquin L, Limanton E, Lagrost C, Morineau D. Thermal, dielectric and electrochemical study of decanoic acidtetrabutylammonium chloride deep eutectic solvent. Comptes Rendus Chimie. 2024;27(2024).

[23] Demmelmayer P, Steiner L, Weber H, Kienberger M. Thymol-Menthol-Based deep eutectic solvent to replace 1–Octanol as modifier in reactive Liquid-Liquid extraction of lactic acid, acetic acid and oxalic acid from pretreated sweet sorghum silage press juice. Sep Purif Technol. 2023;310:123060.

[24] Devi M, Moral R, Thakuria S, Mitra A, Paul S. Hydrophobic deep eutectic solvents as greener substitutes for conventional extraction media: examples and techniques. ACS Omega. 2023;8(11):9702–28. 36969397 10.1021/acsomega.2c07684 PMC10034849

[25] Zhang Y, Qiao Q, Abbas UL, Liu J, Zheng Y, Jones C, et al. Lignin derived hydrophobic deep eutectic solvents as sustainable extractants. J Clean Prod. 2023;388:135808.

[26] Liu C, Ullah A, Gao X, Shi J. Synergistic ball Milling–Enzymatic pretreatment of brewer’s spent grains to improve volatile fatty acid production through thermophilic anaerobic fermentation. Processes. 2023;11(6):1648.

[27] Zhang X, Wang J, Zhang Y, Qing W, Lansing S, Shi J, et al. Anhydrous volatile fatty acid extraction through omniphobic membranes by hydrophobic deep eutectic solvents: mechanistic Understanding and future perspective. Water Res. 2024;257:121654. 38701552 10.1016/j.watres.2024.121654

[28] Abraham MJ, Murtola T, Schulz R, Páll S, Smith JC, Hess B, et al. GROMACS: high performance molecular simulations through multi-level parallelism from laptops to supercomputers. SoftwareX. 2015;1:19–25.

[29] Abbas UL, Qiao Q, Nguyen MT, Shi J, Shao Q. Structure and hydrogen bonds of hydrophobic deep eutectic solvent-aqueous liquid–liquid interfaces. AIChE J. 2021;67(12):e17427.

[30] Abbas UL, Qiao Q, Nguyen MT, Shi J, Shao Q. Molecular dynamics simulations of heterogeneous hydrogen bond environment in hydrophobic deep eutectic solvents. AIChE J. 2021;68(1):e17382.

[31] Robertson MJ, Tirado-Rives J, Jorgensen WL. Improved peptide and protein torsional energetics with the OPLS-AA force field. J Chem Theory Comput. 2015;11(7):3499–509. 26190950 10.1021/acs.jctc.5b00356 PMC4504185

[32] Dodda LS, Cabeza de Vaca I, Tirado-Rives J, Jorgensen WL. LigParGen web server: an automatic OPLS-AA parameter generator for organic ligands. Nucleic Acids Res. 2017;45(W1):W331–6. 28444340 10.1093/nar/gkx312 PMC5793816

[33] Dodda LS, Vilseck JZ, Tirado-Rives J, Jorgensen WL. 1.14* CM1A-LBCC: localized bond-charge corrected CM1A charges for condensed-phase simulations. J Phys Chem B. 2017;121(15):3864–70. 28224794 10.1021/acs.jpcb.7b00272 PMC5813481

[34] Jorgensen WL, Tirado-Rives J. Potential energy functions for atomic-level simulations of water and organic and biomolecular systems. Proceedings of the National Academy of Sciences. 2005;102(19):6665-70. 10.1073/pnas.0408037102 PMC1100738 15870211

[35] Berendsen HJ, Van Postma Jv WF, DiNola A, Haak JR. Molecular dynamics with coupling to an external bath. J Chem Phys. 1984;81(8):3684–90.

[36] Parrinello M, Rahman A. Polymorphic transitions in single crystals: A new molecular dynamics method. J Appl Phys. 1981;52(12):7182–90.

[37] Nosé S, Klein M. Constant pressure molecular dynamics for molecular systems. Mol Phys. 1983;50(5):1055–76.

[38] Darden T, York D, Pedersen L. Particle mesh ewald: an N⋅ log (N) method for Ewald sums in large systems. J Chem Phys. 1993;98(12):10089–92.

[39] Hess B, Bekker H, Berendsen HJ, Fraaije JG. LINCS: A linear constraint solver for molecular simulations. J Comput Chem. 1997;18(12):1463–72.

[40] Yalkowsky SH, He Y, Jain P. Handbook of aqueous solubility data. CRC; 2016.

[41] Swanson WS. Sorption of organic materials by pure clay minerals. 1963.

[42] Rizzioli F, Battista F, Bolzonella D, Frison N. Volatile fatty acid recovery from anaerobic fermentate: focusing on adsorption and desorption performances. Ind Eng Chem Res. 2021;60(37):13701–9.

[43] Clark ME, Bear JL. Metal ion complex formation in non-aqueous solvents: formation constants of Cu(II) and Yb(III) isobutyrate and α-hydroxyisobutyrate. J Inorg Nucl Chem. 1970;32(11):3569–74.

[44] Zhang Y, Hunter JR, Ullah A, Shao Q, Shi J. Lignin derived hydrophobic deep eutectic solvents for the extraction of nanoplastics from water. J Hazard Mater. 2024;467:133695. 38341895 10.1016/j.jhazmat.2024.133695

[45] Yamamoto Y, Goto M, Hanada T, Moriyama T, Procter M. inventorsMethod for recycling hydrophobic deep eutectic solvent and nickel leaching method 2024.

[46] Macário I, Oliveira H, Menezes A, Ventura S, Pereira J, Gonçalves A, et al. Cytotoxicity profiling of deep eutectic solvents to human skin cells. Sci Rep. 2019;9(1):1–9. 30850631 10.1038/s41598-019-39910-y PMC6408470

[47] Panda DK, Bhargava B. Molecular dynamics investigation of non-ionic deep eutectic solvents. J Mol Graphics Modelling. 2022;113:108152. 10.1016/j.jmgm.2022.108152 35202956

[48] Malik A, Kashyap HK. Heterogeneity in hydrophobic deep eutectic solvents: SAXS Prepeak and local environments. Phys Chem Chem Phys. 2021;23(6):3915–24. 33543176 10.1039/d0cp05407k

[49] Mokhtarpour M, Shekaari H, Zafarani-Moattar MT, Golgoun S. Solubility and solvation behavior of some drugs in choline based deep eutectic solvents at different temperatures. J Mol Liq. 2020;297.

[50] Paul N, Naik PK, Ribeiro BD, Gooh Pattader PS, Marrucho IM, Banerjee T. Molecular dynamics insights and water stability of hydrophobic deep eutectic solvents aided extraction of nitenpyram from an aqueous environment. J Phys Chem B. 2020;124(34):7405–20. 32706582 10.1021/acs.jpcb.0c03647

[51] Ashworth CR, Matthews RP, Welton T, Hunt PA. Doubly ionic hydrogen bond interactions within the choline chloride-urea deep eutectic solvent. Phys Chem Chem Phys. 2016;18(27):18145–60. 27328990 10.1039/c6cp02815b

[52] D’Oria E, Novoa JJ. Cation-anion hydrogen bonds: a new class of hydrogen bonds that extends their strength beyond the covalent limit. A theoretical characterization. J Phys Chem A. 2011;115(45):13114–23. 21942671 10.1021/jp205176e

[53] Boo C, Lee J, Elimelech M. Omniphobic polyvinylidene fluoride (PVDF) membrane for desalination of shale gas produced water by membrane distillation. Environ Sci Technol. 2016;50(22):12275–82. 27762141 10.1021/acs.est.6b03882

[54] Li C, Li X, Du X, Zhang Y, Wang W, Tong T, et al. Elucidating the Trade-off between membrane wetting resistance and water vapor flux in membrane distillation. Environ Sci Technol. 2020;54(16):10333–41. 32702974 10.1021/acs.est.0c02547

[55] Ni T, Zhao S, Kong L, Lin J. Omniphobic membranes: fundamentals, materials, and applications. In: Li X, Lin J, Zhao S, editors. Advances in functional separation membranes. The Royal Society of Chemistry; 2021. p. 0.

[56] Zhu P, Kong T, Tang X, Wang L. Well-defined porous membranes for robust omniphobic surfaces via microfluidic emulsion templating. Nat Commun. 2017;8(1):15823. 28604698 10.1038/ncomms15823 PMC5472779

