# Supplementary material for: Volatile fatty acids recovery from thermophilic acidogenic fermentation using hydrophobic deep eutectic solvents
Source: J Biol Eng. 2025 Jul 31;19:73. doi: 10.1186/s13036-025-00544-6 (PMC12315322; doi:10.1186/s13036-025-00544-6)
Supplement: Supplementary file 1 — Supplementary Material 1 [file 13036_2025_544_MOESM1_ESM.docx]

*Appendix*

**Volatile Fatty Acids Recovery from Thermophilic Acidogenic Fermentation using Hydrophobic Deep Eutectic Solvents**

Can Liu^†^, Xueyao Zhang^‡^, Qi Qiao^§^, Zhiwu Wang^‡^, Qing Shao^§^, Jian Shi^†, *^

^†^ Biosystems and Agricultural Engineering, University of Kentucky, Lexington, KY 40546, USA

^‡^ Department of Biological Systems Engineering, Virginia Polytechnic Institute and State University, Blacksburg, VA 24061, USA

^§^ Chemical and Materials Engineering, University of Kentucky, Lexington, KY 40546, USA

**Table A.1:** DES systems built and calculated for molecular simulations.

**Table A.2.** Force field parameters for tetraoctylammonium bromide (TOAB), decanoic acid (DA), Thymol (Thy), 2,6-dimethoxyphenol (DMP), ammonia butyrate (But), and butyric acid (BA) molecules/formula units

**Table A.3.** Pre-experimental VFAs concentrations (mM) in the synthetic mixture (S-a) before and after extraction at 3, 6, and 12 hours.

**Table A.4.** VFAs concentrations before and after extraction for the artificial fermentate (A-n) and filtered real fermentate (F-n), extractant: TBAC/DA (1:2), t=3 h.

**Table A.1.** DES systems built and calculated for molecular simulations (TOAB: tetraoctylammonium bromide, DA: decanoic acid, Thy: thymol, DMP: 2,6-dimethoxyphenol, But^+^: butyrate anion, NH_4_^+^: ammonium cation, and BA: butyric acid).

| Name | Components and number of each molecule/formula unit | | | | | | | | |
| --- | --- | --- | --- | --- | --- | --- | --- | --- | --- |
|  | H_2_O | Thy | DMP | TOA^+^ | Br^-^ | DA | BA | But^-^ | NH_4_^+^ |
| H_2_O_25_1BA | 2165 | - | - | - | - | - | 1 | - | - |
| H_2_O_55_1BA | 2165 | - | - | - | - | - | 1 | - | - |
| Thy_DMP12_25 | - | 100 | 200 | - | - | - | - | - | - |
| Thy_DMP12_55 | - | 100 | 200 | - | - | - | - | - | - |
| Thy_DMP12_25_1BA | - | 100 | 200 | - | - | - | 1 | - | - |
| Thy_DMP12_55_1BA | - | 100 | 200 | - | - | - | 1 | - | - |
| TOAB_DA12_55_H_2_O | 5322 | - | - | 100 | 100 | 200 | - | - | - |
| Thy_DMP12_55_ H_2_O | 2165 | 100 | 200 | - | - | - | - | - | - |
| Thy_DMP12_55_ H_2_O+ | 4330 | 100 | 200 | - | - | - | - | - | - |
| Thy_DMP12_55_ 1But | - | 100 | 200 | - | - | - | - | 1 | 1 |
| TOAB_DA12_55 | - | - | - | 100 | 100 | 200 | - | - | - |
| TOAB_DA12_55_BA | - | - | - | 100 | 100 | 200 | 1 | - | - |
| TOAB_DA12_55_But | - | - | - | 100 | 100 | 200 | - | 1 | 1 |

**Table A.2.** Force field parameters for tetraoctylammonium bromide (TOAB), decanoic acid (DA), Thymol (Thy), 2,6-dimethoxyphenol (DMP), ammonia butyrate (But), and butyric acid (BA) molecules/formula units

| TOAB-TOA^+^ | | | |
| --- | --- | --- | --- |
| Atom | σ (nm) | ε (kJ/mol) | q (e) |
| C00 | 0.3500 | 0.2761 | -0.2108 |
| C01 | 0.3500 | 0.2761 | -0.1563 |
| C02 | 0.3500 | 0.2761 | -0.1564 |
| C03 | 0.3500 | 0.2761 | -0.1561 |
| C04 | 0.3500 | 0.2761 | -0.1596 |
| C05 | 0.3500 | 0.2761 | -0.149 |
| C06 | 0.3500 | 0.2761 | -0.1974 |
| C07 | 0.3500 | 0.2761 | -0.0548 |
| N08 | 0.3250 | 0.7113 | -0.1225 |
| C09 | 0.3500 | 0.2761 | -0.0738 |
| C0A | 0.3500 | 0.2761 | -0.1836 |
| C0B | 0.3500 | 0.2761 | -0.1549 |
| C0C | 0.3500 | 0.2761 | -0.1561 |
| C0D | 0.3500 | 0.2761 | -0.1561 |
| C0E | 0.3500 | 0.2761 | -0.1561 |
| C0F | 0.3500 | 0.2761 | -0.1566 |
| C0G | 0.3500 | 0.2761 | -0.2107 |
| C0H | 0.3500 | 0.2761 | -0.0706 |
| C0I | 0.3500 | 0.2761 | -0.1898 |
| C0J | 0.3500 | 0.2761 | -0.152 |
| C0K | 0.3500 | 0.2761 | -0.1587 |
| C0M | 0.3500 | 0.2761 | -0.1555 |
| C0N | 0.3500 | 0.2761 | -0.1569 |
| C0O | 0.3500 | 0.2761 | -0.1563 |
| C0P | 0.3500 | 0.2761 | -0.2107 |
| C0Q | 0.3500 | 0.2761 | -0.0647 |
| C0R | 0.3500 | 0.2761 | -0.1954 |
| C0S | 0.3500 | 0.2761 | -0.1501 |
| C0T | 0.3500 | 0.2761 | -0.1599 |
| C0U | 0.3500 | 0.2761 | -0.1569 |
| C0V | 0.3500 | 0.2761 | -0.1569 |
| C0W | 0.3500 | 0.2761 | -0.1575 |
| C0X | 0.3500 | 0.2761 | -0.2104 |
| H0Y | 0.2500 | 0.1255 | 0.0759 |
| H0Z | 0.2500 | 0.1255 | 0.0759 |
| H10 | 0.2500 | 0.1255 | 0.0759 |
| H11 | 0.2500 | 0.1255 | 0.0812 |
| H12 | 0.2500 | 0.1255 | 0.0812 |
| H13 | 0.2500 | 0.1255 | 0.0797 |
| H14 | 0.2500 | 0.1255 | 0.0797 |
| H15 | 0.2500 | 0.1255 | 0.086 |
| H16 | 0.2500 | 0.1255 | 0.086 |
| H17 | 0.2500 | 0.1255 | 0.0797 |
| H18 | 0.2500 | 0.1255 | 0.0797 |
| H19 | 0.2500 | 0.1255 | 0.0947 |
| H1A | 0.2500 | 0.1255 | 0.0947 |
| H1B | 0.2500 | 0.1255 | 0.0999 |
| H1C | 0.2500 | 0.1255 | 0.0999 |
| H1D | 0.2500 | 0.1255 | 0.1275 |
| H1E | 0.2500 | 0.1255 | 0.1275 |
| H1F | 0.2500 | 0.1255 | 0.1286 |
| H1G | 0.2500 | 0.1255 | 0.1286 |
| H1H | 0.2500 | 0.1255 | 0.0988 |
| H1I | 0.2500 | 0.1255 | 0.0988 |
| H1J | 0.2500 | 0.1255 | 0.091 |
| H1K | 0.2500 | 0.1255 | 0.091 |
| H1M | 0.2500 | 0.1255 | 0.0838 |
| H1N | 0.2500 | 0.1255 | 0.0838 |
| H1O | 0.2500 | 0.1255 | 0.0838 |
| H1P | 0.2500 | 0.1255 | 0.0838 |
| H1Q | 0.2500 | 0.1255 | 0.0811 |
| H1R | 0.2500 | 0.1255 | 0.0811 |
| H1S | 0.2500 | 0.1255 | 0.0803 |
| H1T | 0.2500 | 0.1255 | 0.0803 |
| H1U | 0.2500 | 0.1255 | 0.0761 |
| H1V | 0.2500 | 0.1255 | 0.0761 |
| H1W | 0.2500 | 0.1255 | 0.0761 |
| H1X | 0.2500 | 0.1255 | 0.1298 |
| H1Y | 0.2500 | 0.1255 | 0.1298 |
| H1Z | 0.2500 | 0.1255 | 0.098 |
| H20 | 0.2500 | 0.1255 | 0.098 |
| H21 | 0.2500 | 0.1255 | 0.0939 |
| H22 | 0.2500 | 0.1255 | 0.0939 |
| H23 | 0.2500 | 0.1255 | 0.082 |
| H24 | 0.2500 | 0.1255 | 0.082 |
| H25 | 0.2500 | 0.1255 | 0.0856 |
| H26 | 0.2500 | 0.1255 | 0.0856 |
| H27 | 0.2500 | 0.1255 | 0.0803 |
| H28 | 0.2500 | 0.1255 | 0.0803 |
| H29 | 0.2500 | 0.1255 | 0.0811 |
| H2A | 0.2500 | 0.1255 | 0.0811 |
| H2B | 0.2500 | 0.1255 | 0.0759 |
| H2C | 0.2500 | 0.1255 | 0.0759 |
| H2D | 0.2500 | 0.1255 | 0.0759 |
| H2E | 0.2500 | 0.1255 | 0.1292 |
| H2F | 0.2500 | 0.1255 | 0.1292 |
| H2G | 0.2500 | 0.1255 | 0.1006 |
| H2H | 0.2500 | 0.1255 | 0.1006 |
| H2I | 0.2500 | 0.1255 | 0.0948 |
| H2J | 0.2500 | 0.1255 | 0.0948 |
| H2K | 0.2500 | 0.1255 | 0.0821 |
| H2M | 0.2500 | 0.1255 | 0.0821 |
| H2N | 0.2500 | 0.1255 | 0.0855 |
| H2O | 0.2500 | 0.1255 | 0.0855 |
| H2P | 0.2500 | 0.1255 | 0.0809 |
| H2Q | 0.2500 | 0.1255 | 0.0809 |
| H2R | 0.2500 | 0.1255 | 0.0808 |
| H2S | 0.2500 | 0.1255 | 0.0808 |
| H2T | 0.2500 | 0.1255 | 0.076 |
| H2U | 0.2500 | 0.1255 | 0.076 |
| H2V | 0.2500 | 0.1255 | 0.076 |
|  |  |  |  |
| TOAB-Br^-^ | | | |
| Atom | σ (nm) | ε (kJ/mol) | q (e) |
| Br | 0.4624 | 0.3766 | -1.000 |
|  |  |  |  |
| DA | | | |
| Atom | σ (nm) | ε (kJ/mol) | q (e) |
| C00 | 0.3500 | 0.2761 | -0.2389 |
| C01 | 0.3500 | 0.2761 | -0.1793 |
| C02 | 0.3500 | 0.2761 | -0.1782 |
| C03 | 0.3500 | 0.2761 | -0.1792 |
| C04 | 0.3500 | 0.2761 | -0.1785 |
| C05 | 0.3500 | 0.2761 | -0.1782 |
| C06 | 0.3500 | 0.2761 | -0.1795 |
| C07 | 0.3500 | 0.2761 | -0.1706 |
| C08 | 0.3500 | 0.2761 | -0.2606 |
| C09 | 0.3550 | 0.2929 | 0.4599 |
| O0A | 0.2960 | 0.8786 | -0.3943 |
| O0B | 0.3120 | 0.7113 | -0.5602 |
| H0C | 0.2500 | 0.1255 | 0.081 |
| H0D | 0.2500 | 0.1255 | 0.081 |
| H0E | 0.2500 | 0.1255 | 0.081 |
| H0F | 0.2500 | 0.1255 | 0.0889 |
| H0G | 0.2500 | 0.1255 | 0.0889 |
| H0H | 0.2500 | 0.1255 | 0.0892 |
| H0I | 0.2500 | 0.1255 | 0.0892 |
| H0J | 0.2500 | 0.1255 | 0.0907 |
| H0K | 0.2500 | 0.1255 | 0.0907 |
| H0M | 0.2500 | 0.1255 | 0.089 |
| H0N | 0.2500 | 0.1255 | 0.089 |
| H0O | 0.2500 | 0.1255 | 0.0937 |
| H0P | 0.2500 | 0.1255 | 0.0937 |
| H0Q | 0.2500 | 0.1255 | 0.0881 |
| H0R | 0.2500 | 0.1255 | 0.0881 |
| H0S | 0.2500 | 0.1255 | 0.1109 |
| H0T | 0.2500 | 0.1255 | 0.1109 |
| H0U | 0.2500 | 0.1255 | 0.1212 |
| H0V | 0.2500 | 0.1255 | 0.1212 |
| H0W | 0.0000 | 0.0000 | 0.4512 |
|  |  |  |  |
| Thy | | | |
| Atom | σ (nm) | ε (kJ/mol) | q (e) |
| C00 | 0.3500 | 0.2761 | -0.1996 |
| C01 | 0.3550 | 0.2929 | -0.0404 |
| C02 | 0.3550 | 0.2929 | -0.2496 |
| C03 | 0.3550 | 0.2929 | 0.1366 |
| C04 | 0.3550 | 0.2929 | -0.1052 |
| C05 | 0.3550 | 0.2929 | -0.102 |
| C06 | 0.3550 | 0.2929 | -0.1913 |
| C07 | 0.3500 | 0.2761 | -0.0617 |
| C08 | 0.3500 | 0.2761 | -0.2287 |
| C09 | 0.3500 | 0.2761 | -0.2341 |
| O0A | 0.3120 | 0.7113 | -0.5019 |
| H0B | 0.2500 | 0.1255 | 0.0947 |
| H0C | 0.2500 | 0.1255 | 0.0947 |
| H0D | 0.2500 | 0.1255 | 0.0947 |
| H0E | 0.2420 | 0.1255 | 0.1488 |
| H0F | 0.2420 | 0.1255 | 0.1536 |
| H0G | 0.2420 | 0.1255 | 0.1504 |
| H0H | 0.2500 | 0.1255 | 0.1162 |
| H0I | 0.2500 | 0.1255 | 0.0837 |
| H0J | 0.2500 | 0.1255 | 0.0837 |
| H0K | 0.2500 | 0.1255 | 0.0837 |
| H0M | 0.2500 | 0.1255 | 0.0823 |
| H0N | 0.2500 | 0.1255 | 0.0823 |
| H0O | 0.2500 | 0.1255 | 0.0823 |
| H0P | 0.0000 | 0.0000 | 0.4268 |
| DMP | | | |
| Atom | σ (nm) | ε (kJ/mol) | q (e) |
| C00 | 0.3500 | 0.2761 | -0.0406 |
| O01 | 0.2900 | 0.5858 | -0.3334 |
| C02 | 0.3550 | 0.2929 | 0.0599 |
| C03 | 0.3550 | 0.2929 | 0.2618 |
| C04 | 0.3550 | 0.2929 | 0.1276 |
| C05 | 0.3550 | 0.2929 | -0.1667 |
| C06 | 0.3550 | 0.2929 | -0.1134 |
| C07 | 0.3550 | 0.2929 | -0.2059 |
| O08 | 0.2900 | 0.5858 | -0.2974 |
| C09 | 0.3500 | 0.2761 | -0.0493 |
| O0A | 0.3120 | 0.7113 | -0.7216 |
| H0B | 0.2500 | 0.1255 | 0.0968 |
| H0C | 0.2500 | 0.1255 | 0.0968 |
| H0D | 0.2500 | 0.1255 | 0.0968 |
| H0E | 0.2420 | 0.1255 | 0.1604 |
| H0F | 0.2420 | 0.1255 | 0.1466 |
| H0G | 0.2420 | 0.1255 | 0.1455 |
| H0H | 0.2500 | 0.1255 | 0.0964 |
| H0I | 0.2500 | 0.1255 | 0.0964 |
| H0J | 0.2500 | 0.1255 | 0.0964 |
| H0K | 0.0000 | 0.0000 | 0.4469 |
|  |  |  |  |
| But-NH_4_^+^ | | | |
| Atom | σ (nm) | ε (kJ/mol) | q (e) |
| N00 | 0.3250 | 0.7113 | -0.5176 |
| H01 | 0.0000 | 0.0000 | 0.3794 |
| H02 | 0.0000 | 0.0000 | 0.3794 |
| H03 | 0.0000 | 0.0000 | 0.3794 |
| H04 | 0.0000 | 0.0000 | 0.3794 |
|  |  |  |  |
| But-But^-^ | | | |
| Atom | σ (nm) | ε (kJ/mol) | q (e) |
| C00 | 3.50E-01 | 2.76E-01 | -0.2085 |
| C01 | 3.50E-01 | 2.76E-01 | -0.1371 |
| C02 | 3.50E-01 | 2.76E-01 | -0.2324 |
| C03 | 3.55E-01 | 2.93E-01 | 0.4278 |
| O04 | 2.96E-01 | 8.79E-01 | -0.6323 |
| O05 | 2.96E-01 | 8.79E-01 | -0.6323 |
| H06 | 2.50E-01 | 1.26E-01 | 0.0482 |
| H07 | 2.50E-01 | 1.26E-01 | 0.0482 |
| H08 | 2.50E-01 | 1.26E-01 | 0.0482 |
| H09 | 2.50E-01 | 1.26E-01 | 0.0768 |
| H0A | 2.50E-01 | 1.26E-01 | 0.0768 |
| H0B | 2.50E-01 | 1.26E-01 | 0.0583 |
| H0C | 2.50E-01 | 1.26E-01 | 0.0583 |
|  |  |  |  |
| BA | | | |
| Atom | σ (nm) | ε (kJ/mol) | q (e) |
| C00 | 0.3500 | 0.2761 | -0.2423 |
| C01 | 0.3500 | 0.2761 | -0.1695 |
| C02 | 0.3500 | 0.2761 | -0.2602 |
| C03 | 0.3550 | 0.2929 | 0.4597 |
| O04 | 0.2960 | 0.8786 | -0.3947 |
| O05 | 0.3120 | 0.7113 | -0.5604 |
| H06 | 0.2500 | 0.1255 | 0.0847 |
| H07 | 0.2500 | 0.1255 | 0.0847 |
| H08 | 0.2500 | 0.1255 | 0.0847 |
| H09 | 0.2500 | 0.1255 | 0.11 |
| H0A | 0.2500 | 0.1255 | 0.11 |
| H0B | 0.2500 | 0.1255 | 0.121 |
| H0C | 0.2500 | 0.1255 | 0.121 |
| H0D | 0.0000 | 0.0000 | 0.4513 |

**Table A.3.** Pre-experimental VFAs concentrations (mM) in the synthetic mixture (S-a) before and after extraction at 3, 6, and 12 hours.

| Time | Acetic acid | Propionic acid | Isobutyric acid | Butyric acid | Valeric acid |
| --- | --- | --- | --- | --- | --- |
| 0 h | 9.92 | 12.53 | 10.18 | 9.69 | 10.31 |
| Men/Thy (1:1) | | | | | |
| 3 h | 7.87 | 5.63 | 1.73 | 1.81 | 0.60 |
| 6 h | 7.69 | 5.27 | 1.58 | 1.62 | 0.50 |
| 12 h | 7.76 | 5.42 | 1.67 | 1.69 | 0.52 |
| Men/DA (2:1) | | | | | |
| 3 h | 8.15 | 5.82 | 1.87 | 1.93 | 0.61 |
| 6 h | 8.12 | 5.58 | 1.66 | 1.81 | 0.54 |
| 12 h | 7.83 | 5.58 | 1.61 | 1.75 | 0.54 |
| TBAC/DA (1:2) | | | | | |
| 3 h | 6.39 | 5.02 | 2.05 | 2.01 | 0.81 |
| 6 h | 6.37 | 4.97 | 2.00 | 1.94 | 0.78 |
| 12 h | 6.50 | 5.24 | 2.05 | 2.02 | 0.85 |
| Thy/DMP (1:2) | | | | | |
| 3 h | 7.30 | 5.48 | 2.24 | 2.05 | 0.73 |
| 6 h | 7.16 | 5.28 | 2.10 | 1.94 | 0.72 |
| 12 h | 7.17 | 5.41 | 2.33 | 1.96 | 0.69 |

Note: This pre-experimental test was conducted prior to the main experiments to determine appropriate extraction conditions. No replicates were performed for these data.

**Table A.4.** VFAs concentrations before and after extraction for the artificial fermentate (A-n) and filtered real fermentate (F-n), extractant: TBAC/DA (1:2), t=3 h.

|  | Acetic acid | Propionic acid | Isobutyric acid | Butyric acid | Valeric acid |
| --- | --- | --- | --- | --- | --- |
| A-n Initial conc. | 255.68±1.35 | 41.25±0.27 | 3.96±0.09 | 120.44±0.70 | 2.88±0.15 |
| A-n Equil. conc. | 140.65±0.19 | 14.13±0.11 | 0.65±0.03 | 19.63±0.08 | 0.24±0.00 |
| F-n Initial conc. | 131.01±0.12 | 42.94±0.42 | 1.36±0.07 | 111.12±0.43 | 0.25±0.01 |
| F-n Equil. conc. | 72.32±3.37 | 16.57±0.92 | 0.45±0.04 | 23.27±1.11 | 0.04±0.01 |
